# COVID-19—What Price Do Children Pay? An Analysis of Economic and Social Policy Factors

**DOI:** 10.3390/ijerph19137604

**Published:** 2022-06-21

**Authors:** Stephanie Lange, Claire-Marie Altrock, Emily Gossmann, Jörg M. Fegert, Andreas Jud

**Affiliations:** 1 Clinic for Child and Adolescent Psychiatry/Psychotherapy, University Hospital of Ulm, Steinhövelstraße 5, 89075 Ulm, Germany; claire-marie.altrock@uni-ulm.de (C.-M.A.); emily.gossmann@uniklinik-ulm.de (E.G.); joerg.fegert@uniklinik-ulm.de (J.M.F.); andreas.jud@uniklinik-ulm.de (A.J.); 2 Competence Area Mental Health Prevention in the Competence Network Preventive Medicine Baden-Württemberg, Clinic for Child and Adolescent Psychiatry/Psychotherapy, University Hospital of Ulm, Steinhövelstraße 5, 89075 Ulm, Germany; 3 Competence Center Child Abuse and Neglect Baden-Württemberg, Clinic for Child and Adolescent Psychiatry/Psychotherapy, University Hospital of Ulm, Steinhövelstraße 5, 89075 Ulm, Germany; 4 School of Social Work, Lucerne University of Applied Sciences and Arts, 6000 Lucerne, Switzerland

**Keywords:** children and adolescents, COVID-19, economic consequences, social conflicts, mental health

## Abstract

Numerous studies have addressed the indirect consequences of the COVID-19 pandemic for children such as social isolation or increases in reported child maltreatment. Research on the economic and sociopolitical consequences is scarce as they can only be evaluated with a time lag. To improve our understanding of future, long-term developments in the context of the COVID-19 pandemic, we gathered findings from the still unexploited empirical literature on the aftermath of earlier pandemics, epidemics, and other infectious disease outbreaks. On top of this, we scrutinized research on past economic crises to interpret the link between changes in the economy and the health of children. Many of the side effects of battling the spread of the current pandemic, such as school closures, the stigma of infection, or conflicts about vaccines, are not novel and have already been documented in connection with previous infectious disease outbreaks. Results highlight that changes in the financial situation of families and socio-political challenges affect the situation and daily routine of children and youth in the long term. In consequence, the already pronounced socioeconomic inequalities will likely further increase. On top of this, due to reduced revenues, child protective services are likely to face challenges in the availability of human and financial resources.

## 1. Introduction

At the start of the COVID-19 pandemic, direct consequences of the lockdown measures for children and adolescents were observed, such as an increase in family violence and social isolation [1,2], but longer-term social and economic effects show up only in a later phase of the pandemic or afterward. In Germany, for example, a report from the Federal Statistical Office of Germany revealed that the COVID-19 pandemic had negative financial effects, in particular on low-income earners, low-skilled people, and single parents [3]. This finding is particularly serious economically, socially, and politically because, compared to the 1990s, today, more persons live in poverty and poverty is more difficult to escape [3]. A German survey of more than 2000 low-income families in November 2020 found that approximately 30% of families had less money at the end of the month than they did before the pandemic [4]. For some of the survey respondents in this high-income country, this meant that they could not pay bills, and 7% even had to do without meals. Especially affected were families that lived below the poverty line and had no option to work from home [4]. Thus, school children from families experiencing poverty often face multiple challenges due to the pandemic: In addition to financial insecurity, there are inequalities for example in remote school instruction, as the families may not own sufficient digital devices. This represents a further disadvantage regarding equal opportunities for educational success. Beyond that, low-income earners did not have the same opportunity to work from home during the pandemic [3]. It is also reasonable to assume that parents with low incomes cannot support their children in remote learning at home as much as parents with higher socioeconomic status and the option to work from home can.

In addition, during the pandemic, the life situations of children and families with supposedly ‘secure’ social and financial circumstances in the past can change. With an aggregation of factors that lead to financial difficulties, such as reduced income due to short-time allowances, unemployment, and rising rent levels, there is a danger that in the long term, families that before the pandemic had sufficient financial reserves will need support. A survey conducted by the Leibniz Institute for Economic Research found that about a third of the households surveyed in high-income Germany expected future pandemic-related financial losses [5].

The pandemic also had a strong impact on education and children’s social lives. School closures occurred as an intervention in the COVID-19 pandemic in more than 190 countries around the world to prevent further infections. Globally, over 94% of students were affected [6]. Studies not only confirm school closures’ impact on education [7] but also find them to have an impact on child behavior [8] and child protection outcomes [9]. For past pandemics or epidemics, however, little research focus has been on the long-term influences on the education of affected children. In the context of the COVID-19 pandemic, simulations can currently be used primarily to assess long-term consequences (e.g., [7]).

From a social point of view, pandemic-related changes in everyday situations have opened up new perspectives and chances for the family environment that can also enrich daily life in the future. Both parents and children see spending more time with the family as a positive aspect. Although children felt burdened by restrictions on contact with friends, they found new ways to communicate with each other via digital media [10]. In addition to daily contact, free-time activities also shifted to the Internet, so that children did not have to do without these activities entirely [10].

However, already after the first months of the pandemic, children reported having more arguments in the family, and “parents reported that arguments with their children escalated more often” [11] (p. 828). Besides family conflicts, policy decisions and measures as the pandemic continued have led to numerous social conflicts of interest that also directly affect children and adolescents in their everyday lives. Children can be caught up in the tensions of conflicts over accepting COVID-19 measures between parents, friends, school peers, teachers, or leaders of recreational activities. It is not so much the regulations and measures themselves that put pressure on children but rather acceptance and expectations concerning the measures by adults and peers. Moreover, if children or their family members become ill with COVID-19, this can result in exclusion, stigmatization, and conflict situations.

The waves of infection brought similar public health measures and contrary opinions on vaccination in many countries. With the increasing infection rates and the discussion on vaccinating children, school leaders in Germany, for example, feared there would be increasing conflicts between parents, school children, and teachers regarding quarantine regulations and willingness to be vaccinated [12]. When contrary to school rules parents forbid their children to wear masks, terms such as instrumentalization of children and loyalty conflict for children are used [13]. Depending on whether homeschooling was common in a country already before the pandemic or not, local regulations for children and adolescents can have a direct effect on their school participation. This is evident in two examples in Germany; children in Bavaria whose parents did not allow them to be tested for COVID-19 received instruction materials but were not allowed to attend regular classroom instruction [14]. According to the news magazine Der Spiegel (2021), the children were treated like truants, and some families had to pay a fine [15]. In Schleswig-Holstein, in contrast, it is no longer possible for parents to keep their children out of school even if they are against masks or tests [16].

Particularly aggressive parents pose a challenge to the schools beyond the organizational implementation of protection measures [17]. In a German survey, every fifth teacher reported knowing fellow teachers who had been verbally attacked over COVID-19 safety measures [17]. Moreover, COVID-19 protests are increasing worldwide and not infrequently escalating into violence [18], whereby children have also been misused as a ‘protective shield’ [19].

These German examples that are similarly available from other countries around the world show the great extent to which social and economic challenges can have direct effects on children’s everyday lives. In addition, according to a recent study, the psychological well-being of children and adults has worsened since the pandemic, especially in families experiencing multiple hardships [20]. Moreover, all kinds of pandemic-related fears and worries on the part of some family members “can result in enormous stress and psychological distress for all family members” [21] (p. 3). On this basis, increasing social and economic burdens can also change the estimation of risk factors for maltreatment of children and adolescents. An increase in domestic violence is predicted particularly in view of the expected labor market trends [22]. This can lead to a future change in needs in the area of child protective services.

This study aims to identify, in the economic and social policy context, pandemic-related factors that can have long-term effects on children’s everyday lives and health. It will examine whether current developments have already occurred during previous outbreaks or crises and if we could learn from these findings.

For this review, which was developed during the ongoing pandemic, we aimed to continuously respond to new phenomena and trends by searching for evidence on the topic from previous outbreaks of infectious diseases. In accordance with this aim, we were not able to only conduct searches with a predefined combination of terms but were constantly striving to adapt our searches to new developments. Obviously, this approach does not provide a comprehensive systematic review. Keyword search (English and German) in scientific databases (e.g., Web of Science, Google Scholar) was conducted in order to determine whether these or analogous topics had already been addressed in previous disease outbreaks or crises. Afterward, articles that were closest to the current topic were chosen for citation.

As there have not been any pandemics with similar global impacts and challenges in recent decades, there are only a few studies on earlier infectious disease outbreaks and a small number of current studies on which to draw for analysis of social factors. For economic factors, research findings on past economic crises can provide starting points concerning the impact on children’s health and will be included in the analysis.

The selected literature presented does not claim to be exhaustive. It rather falls in line with the scope and method of a scoping review that, in its first step, identifies and analyzes “key characteristics or factors related to a concept” and “knowledge gaps” [23] (p. 2). Nevertheless, with this analysis, we aim to create a basis on which to map out initial contributing factors to children’s and adolescents‘ future support needs, which are difficult to forecast and alert professionals to them. Regardless, a statement about the long-term effects will only be possible at a later date—possibly only in a few years.

## 2. State of Research Related to the COVID-19 Pandemic

The World Health Organization’s (WHO) ‘One Health’ concept is an approach to achieving better public health outcomes by taking the environment in which we live into account [24]. In addition to ensuring food safety and combatting antibiotic resistance, the WHO approach also focuses on the control of zoonoses, infectious diseases that can spread from animals to humans. Examples are HIV, Ebola, and now the novel coronavirus that causes COVID-19 [25]. Previous research has found that changes in the environment, such as climate change, affect the spread of viral infectious diseases [26]. Consequently, the likelihood of future pandemics may increase, and the social and economic impact of infectious diseases will become more important. However, macroeconomic forecasting has persistently underestimated the possibility of a pandemic and thus infectious disease risk [27]. This has led to “underinvestment in preparedness and response to infectious disease crises” [27] (p. 2443). According to Sands et al. [27] (p. 2443), one reason why economists failed to consider economic vulnerability to infectious disease threats was “the absence of readily available and digestible input data”. For future assessment calculations, this may have changed with the start of the COVID-19 pandemic. This means that to relate COVID-19-related economic and social challenges to the body of existing literature, the only studies available for consideration are studies on regional outbreaks of infectious diseases, epidemics, or pandemics in the past and very few current research studies. By definition, an *outbreak* of infectious disease takes place in a specific location and time and with an expected number of occurrences. An outbreak is called an *epidemic* when there is a higher-than-expected, sudden increase in cases of a disease or health event. A *pandemic* is an event in which an infectious disease affects a large number of people and spreads across several countries [28,29].

### 2.1. Social Policy Challenges of Pandemics, Epidemics, and Infectious Disease Outbreaks

A different situation compared to today, especially in the area of caring for children, is revealed by a study that examined the effects of the Spanish Influenza pandemic on the health and mortality rates of children in 1918 and 1919 [30]. Reid found that the mortality of children in the influenza pandemic was dependent not only on children contracting the disease themselves but also on a mother’s health or death.

Research in the social policy area on later infectious disease outbreaks has focused mainly on topics such as vaccination, school closures, or the stigma associated with infection. The analyses frequently take the arising economic consequences into consideration. A majority of the studies available examined the spread of influenza and H1N1 swine-flu virus [31,32,33], Ebola [9,34], or HIV [35,36]. Some studies looked at outbreaks of streptococcal [37] or meningococcal disease [38].

#### 2.1.1. Vaccination

Previous studies on vaccination examined priorities for vaccination interventions [32], the positive impact of public health communication strategies [38], and the socioeconomic and health effects of vaccination programs at schools [31], for example. Basurto-Dávila et al. found that school-based vaccination programs led to higher immunization in children when compared to vaccination in traditional clinic-based settings. This in turn resulted in positive economic effects for society, among other things due to lower costs for work absences of parents caring for ill children [31].

Other studies examined factors influencing vaccination willingness. Findings revealed that both rates of hospitalizations for influenza and influenza vaccination uptake differed depending on socioeconomic status and age [39]. In empirical studies examining reasons for a lack of willingness on the part of parents to have their children vaccinated, parents reported concerns about the safety and effectiveness of vaccines, fear of side effects, and child having already survived the disease [40,41]. Previous studies on vaccination have thus focused mainly on parental or adult involvement and decision making. However, in the course of the current COVID-19 pandemic, the Association of Child and Adolescent Physicians in Germany (BVKJ) noted that for adolescents, school-based vaccination programs also create group pressure that can significantly influence their willingness to be vaccinated [42].

#### 2.1.2. School Closures

Previous studies also examined the economic and social effects of school closures [43] and their effectiveness in stemming the spread of infection [33]. Basurto-Dávila et al. [43] investigated the effect of school closures during the 2009 influenza outbreak in Argentina and found that the majority of families surveyed approved of the school closures but that for households with low socioeconomic status, school closures resulted in significantly higher costs and work absences. Mizumoto et al. [8] examined children’s contact behavior and the effect on parental employment during school closures during the 2009 influenza outbreak in Japan. They found that the frequency with which children left the home during school closures was dependent on age. However, the majority of the survey respondents reported that the school closures had no impact on parental working hours [8].

Regarding content, the areas of vaccination and school closures are directly related to children’s everyday life. However, the existing studies examined mainly economic and social effects and challenges for parents. There are few studies that look at the direct effects on children. A literature review by Villegas et al. [9] for example studied the effect of health-related school closures on selected child protection outcomes. It included 21 available research studies that dealt with Ebola outbreaks in African countries. The review revealed that school closures had an impact on child labor, adolescent pregnancy, and family violence, for instance. According to Villegas et al., the effects were moderated by individual factors, including gender; certain characteristics of the household and community; and factors at the state level, such as educational opportunities [9].

In total, the joint analysis of studies that looked at the impact of school closures on the well-being and health of children during the pandemic point to negative consequences [44]. In some countries, more depressive symptoms and reduced life satisfaction were recorded. In addition, school closures affected referrals to doctors for child protection and child maltreatment [44].

Using data from 174 countries on past school closures due to various causes, Azevedo et al. [7] created four simulations of the long-term impact of school closures during the COVID-19 pandemic on schooling. The scenarios differed in terms of the duration of school closures and policy interventions. All simulations concluded that, across the world, schooling and learning levels will decline sharply. This also has an impact on the subsequent income of the affected children as well as the government. Political interventions can partly mitigate the negative effects [7].

Eyles et al. [45] also highlight that school closures entail very high costs, on the one hand, due to the resources that are not used during the closures, and on the other hand due to the measures to make up for the learning shortfall. It can be assumed that children from disadvantaged families will struggle more with the consequences, as they do not have the necessary support and opportunities to make up for gaps [45].

According to Kaffenberger’s [46] estimate, a three-month school closure results in a one-year learning backlog. If tutoring is offered after the return to the school routine, the backlog can be made up in parts. To do this completely, entire curricula would have to be changed and teachers would have to be trained accordingly [46].

The results underpin the concern that school closures during the COVID-19 pandemic have had negative impacts on children at numerous levels. This holds especially for children who were at risk already prior to the pandemic, such as children with low socioeconomic status. Besides school closures, a lack of child protective services could also have an impact here. The simulations show that it will take a lot of effort to catch up on learning. Interventions need to be planned at the political level and accessed on a large scale.

#### 2.1.3. Stigmatization as a Result of Infection

Stigma in the context of (infectious) diseases means that people with a disease experience negative reactions from others and are excluded from social activities [47]. At the start of the COVID-19 pandemic, in some countries, tourists or immigrants were blamed for the increasing spread of the virus. Once the virus had spread across the entire world, persons infected with COVID-19 and persons who had frequent contact with those with the disease, such as healthcare workers, became the targets of stigma and discrimination [48]. Already early on during the pandemic, a study warned that stigmatization of infected children and families might occur [21].

To assess stigma in the context of COVID-19, reviews on infectious disease outbreaks in the past provide a suitable basis. Previous research on infectious disease-related stigmatization dealt mainly with HIV, tuberculosis [49], and Ebola [50]. Just a few studies looked at the consequences of stigmatization for children and adolescents [51,52].

Haink et al. [35] found that infection with HIV was seen as a bad attribute of the person; persons with HIV were rated as irresponsible and blamed for their own illness. This victim blaming was independent of the blamer’s level of education but increased with age [9]. There was less stigmatization of persons with lower perceived contagiousness, such as those in medical treatment [36]. In the context of the Ebola outbreak in Sierra Leone, children whose parents had died from the disease were stigmatized and excluded from social interactions [52].

Regarding the current COVID-19 pandemic, Bagcchi [53] reported COVID-19-related attacks on healthcare workers during the ongoing pandemic in India and Mexico, and doctors and nurses were denied access to public transportation. A study on COVID-19 survivors in India in August 2020 found that 98% of the survivors had experienced at least one incident of stigmatization [54]. Already for earlier disease outbreaks, reasons named for stigma related to infectious diseases were a lack of knowledge about the illness or an aversion to the unknown [35]. This can be observed also in the course of the current pandemic [55]. In 2020, vaccination was not available for the majority of the population, and there were no effective treatment options for COVID-19; infected persons and survivors were avoided. They reported the loss of friends, insults, and social isolation, which frequently affected their children as well [54].

### 2.2. Economic Effects of Pandemics, Epidemics, and Infectious Disease Outbreaks

Previous research on the economic effects of infectious disease outbreaks examined private and public health costs. Although the medical and health economics conditions are hardly comparable, a study on the 1918 Spanish influenza pandemic found that the cost of operating a hospital in Uppsala, Sweden from 1917 to 1920 doubled due to the outbreak [56]. The authors concluded that today, a similar influenza pandemic would paralyze health care systems.

The worldwide influenza A (H1N1) outbreak in 2009, which the WHO rated as a pandemic [57], also resulted in many countries in a sharp rise in healthcare costs. Suh et al. [58] found total medical costs associated with the pandemic (H1N1) in 2009 increased more than 37-fold compared to earlier outbreaks of seasonal influenza in the Republic of Korea. Further studies have found that especially in lower-middle-income countries, it is particularly difficult to handle the healthcare costs of an outbreak of infectious disease. A systematic literature review by De Francisco et al. [59] provided an overview and differentiated between costs for private households and the public health care system. Huo et al. [60] looked at the outbreak of bird flu (H7N9 virus) in a province in Eastern China and analyzed the individual medical costs for infected persons. Results revealed that costs of hospitalized patients with H7N9 were significant and far surpassed the annual per capita income of the province. Smith et al. [61] also focused on low- and middle-income countries and examined the macroeconomic impact of a mild influenza pandemic. The study found that the cost of mild pandemics was comparatively small. However, “the economic cost of unavoidable [work] absence in the event of an influenza pandemic could be proportionally larger for low-income countries” [61] (p. 1400). To cope with the medical costs of infectious disease outbreaks in developing countries a study by Berry et al. [34] discussed investment in pandemic support funds by industrial countries.

Other studies have modeled the costs of infectious disease outbreaks in dependency on various factors. For instance, Al Kazimi et al. [62] compiled the results of existing studies on the economic costs of natural disasters, terrorist attacks, port closures, and pandemic diseases and compared them to actual and hypothetical economic losses in the United States and quantified them. Factors affecting the costs of a pandemic were working from home, rate of infection, mortality rate, and the stability of Internet connectivity [62]. Prager et al. [63] modeled the economic costs of an influenza outbreak in the United States taking into consideration various conditions such as the presence/absence of vaccinations and differing disease severity.

#### Economic Factors in the Context of Children’s Health

In view of the literature cited in the section above, there are only a few research findings available for outbreaks of infectious disease in the past from which any economic effects of the COVID-19 pandemic on children and adolescents can be derived. Due to the high relevance of the economic impact as outlined above, we now turn to the findings of empirical studies on past recessions and economic crises. Taking these research findings into consideration can provide starting points for identifying factors affecting the health and life circumstances of children in challenging economic times. The factors presented do not represent a definitive list but instead serve as illustrative examples of potential factors.

Table 1 provides a summary of selected study results. For better comparability, the dependent variables in the studies are grouped. Following the WHO’s biopsychosocial model of health, a distinction is made between the physical, mental, and social dimensions of health [64]. Further, child maltreatment is defined as a separate category because of its many and diverse effects on health. Dependent variables that do not fall under the biopsychosocial definition of health or child maltreatment are described according to their operationalization and are not grouped any further.

Literature reviews based on international data found that at an aggregate level, economic crises have a negative impact on children’s physical and mental health and lead to an increase in child maltreatment [21,65,66]. However, during economic crises, whether children’s monetary needs can be fulfilled is country-specific and differs greatly from country to country [67]. A review of the literature by Fegert et al. [68] found that the effects of an economic crisis on the mental health of children and adolescents also differ from country to country.

Analysis of some empirical studies yields a differentiated picture of the equal opportunity of children with different socioeconomic backgrounds. Findings show that especially children that had been disadvantaged financially and socially before economic downturns were affected more severely by an economic crisis [66,69,70]. This was also found in some initial studies on the COVID-19 pandemic [71,72]. The rise in poverty was significant, particularly for families with children [71,72]. On the other hand, it has been found that children born during an economic recession and in families with ex-ante stable economic and social circumstances can profit from the economic downturn from a health perspective: For example, parents had more time to spend with their children and to be concerned about a healthy lifestyle for the family than they did in times of economic prosperity [73]. A study on child health before and after an economic collapse found that Iceland succeeded in warding off the negative effects of an economic crisis and in maintaining children’s physical health and mental well-being then and long-term by quickly instituting governmental support measures, including maintaining the free universal healthcare for pregnant women and children [74]. In contrast, fiscal austerity measures in the area of health care in an economic crisis can result in a decline in children’s physical health and substantively higher childhood mortality [69].

Some of the studies listed in Table 1 provide an overview of some economic factors that can have an impact on child maltreatment. Most of them are based on data from the United States. They show that closely related economic indicators consistently lead to an increase in rates of child maltreatment; child maltreatment incidence rose with economic stresses of recession [75] such as involuntary job losses [76], a rise in the unemployment rate [77,78], and also negative earnings shocks with no sufficient social benefits as supplemental income [79]. Subsequently, also mortgage foreclosure rates [77], neighborhood poverty [80], a decline in the Consumer Sentiment Index [81], and income inequality [82] resulted in an increase in the child maltreatment rate. In addition, [83] (p. 496) found that housing foreclosures give children a feeling of dislocation and chaos and thus “place them at high risk for poor mental health outcomes.”

In summary, analyses of a variety of economic indicators indicate that the economic trend has an impact on children’s health. A direct association has also been found regarding services for children—as documented by Van Dolen et al. [84], a rise in the unemployment rate led to an increase in the number of children’s help-seeking calls to telephone and Internet helplines.

**Table 1:** Overview of studies on the impact of economic factors on child health.

Impact of Economic Factors on Child Health, Child Maltreatment, and Children’s Needs					
Author	Year	Independent Variable	Association	Dependent Variable	Region/Country
Whiteford [85]	1993	Economic crisis	negative impact on	Physical health	Dominican Republic
Bremberg [86]	2003	Proportion of families with low income	no significant impact on	Physical health	Sweden
Bassuk [83]	2010	Mortgage foreclosures	positive impact on	Decreased mental health	USA
Millett et al. [78]	2011	• Unemployment • Food stamps	varying impact depending on state	Child maltreatment	USA
Hong et al. [87]	2011	Economic crisis	positive impact on	Child maltreatment	South Korea
Huang et al. [88]	2011	Recession	positive impact on	Child maltreatment	Ohio, USA
Brooks-Gunn et al. [81]	2013	Consumer Sentiment Index	negative impact on	Child maltreatment	USA
Van Dolen et al. [84]	2013	Unemployment	positive impact on	Children’s help-seeking calls	Netherlands
Frioux et al. [77]	2014	• Unemployment rates • Mortgage foreclosure rates	positive impact on	Child maltreatment	Pennsylvania, USA
Angelini, Mierau [73]	2014	Recession (at the time of childbirth)	positive impact on	Physical and mental health	Europe
Schneider et al. [70]	2015	Economic insecurity	positive impact on	Decreased mental health	USA
Maguire-Jack, Font [80]	2016	(Neighborhood) poverty	positive impact on	Child maltreatment	Ohio, USA
Gunnlaugsson [74]	2016	Economic crisis	(for adapted policies)positive impact on	Physical and mental health	Iceland
Rasella et al. [69]	2018	Austerity measure in health economy	positive impact on	Decreased physical health; child mortality	Brazil
D’Agostino et al. [67]	2019	Economic crisis	varying impact depending on the country	Fulfillment of children’s monetary and non-monetary needs	Europe
Schenck-Fontaine et al. [76]	2020	Involuntary job losses	positive impact on	Child maltreatment	USA
Zhang et al. [82]	2021	Unequal income distribution	positive impact on	Child maltreatment	USA
Cai [79]	2021	Negative earnings shocks (with no sufficient social benefits as supplemental income)	positive impact on	Child maltreatment	Wisconsin, USA

## 3. Support Services in the Context of the Economic Situation

Since the worldwide financial crisis of 2008, approximately 76.5 million children have lived in poverty in the world’s 41 richest countries [89]. Gustavsson and MacEachron [90] found that also after the official end of the recession, unemployment rates remained high in many countries, and revenues in many states decreased, hence funding for state agencies was also reduced. Many states cut funding for health and social services, including child protective services, even though demands for these services increased. This led in many places to a reduction in the services available to affected families and also to unmanageable caseloads at child protective services agencies [90]. There is empirical evidence that poverty increases the likelihood that a family will be involved with child protective services [91]. All this means that economic crises burden children, in that parental income decreases at the same time that institutional safety nets are “increasing incapable of meeting the growing need” [92] (p. 1063). The long-term consequences of the current COVID-19 pandemic for the provision of resources in the area of public child protective services are hard to predict. Many institutional facilities for supporting families may be facing the challenges of not only COVID-19 protection measures but also regarding the future availability of resources. In the OECD countries, the COVID-19 pandemic has generally not affected tax revenues and revenues from social security contributions as much as was the case with economic crises in the past [93]. Still, the effects differ from country to country. In Germany, for instance, area municipalities cover 70–80% of the financing of child protective services [94]. Here, local tax revenues are a substantial source of financing [94]. However, in the course of the pandemic, there has been a sharp decrease in municipal revenues from trade taxes [95]. In addition, there is the risk of a rise in bankruptcies as the pandemic continues and afterward, so that tax revenues could decline even more.

Regarding the current COVID-19 pandemic, Lietzmann and Wenzig [96] found that in Germany in 2020, around 2.8 million children and adolescents under the age of 18 experienced poverty. Although this proportion was similar before the pandemic, children from families experiencing poverty were additionally disadvantaged by the pandemic. For many families affected, the financial situation leads to restrictions on social participation, freedom, and mobility. As for basic material provision, children were found to be better provided for than the adult members of the households; adults seemed to economize more for themselves than for their children [96]. Despite the worsening financial situation of many families, it appears that policy measures introduced by the state during the COVID-19 pandemic have compensated at least temporarily for the income losses: Christl et al. [71] found that the incomes losses and poverty risk were indeed cushioned by the extended short-time working schemes and the one-off payments for children. The average drop in household income was 5% in 2020, but through the policy measures, the reduction in income could be reduced to 0.8%.

## 4. Discussion

This review addresses the impending economic and social policy challenges arising in the COVID-19 pandemic and relates them to existing research on pandemics, epidemics, infectious disease outbreaks, and economic crises in the past. The aim was to examine whether learning effects for today’s situation can be derived from literature on earlier crises. Although the expectation that long-term effects are difficult to predict has been confirmed due to the lack of robust data from a comparable pandemic, there are nevertheless surprising analogies to challenges from earlier times. The review of the literature shows that social policy issues, such as school closures, vaccination willingness, and stigmatization of infected persons, were examined by previous studies on infectious disease outbreaks in the past. Moreover, the effects of economic downturns on children’s health show a unified picture: particular economic costs and social challenges arise mainly in regions with low average income and for target groups with low socioeconomic status.

Furthermore, research studies available already before the COVID-19 pandemic drew attention to hypothetically arising social, economic, and health economy effects of an infectious disease outbreak. Many of these predicted consequences were confirmed in earlier pandemics. Still, the long-term impact in particular of the arising and newly developing social conflicts that are in part also due to the effects of the economic developments, is unclear—as is the further course of the pandemic itself.

Numerous examples presented in the Introduction section above highlight the tensions of conflicts of interest that children and adolescents can be exposed to when coping with everyday life. The first study on the effect of wearing masks on children’s health [97] provides also another approach; in addition to the physical effects of wearing masks, the study examined also the psychological effects, which are closely associated with the social conflicts of interests described above. Schwarz et al. [97] found that 25.3% of children of the parents surveyed had developed new fears, including fear of stigmatization in the social environment for wearing or not wearing a mask. They also reported children’s fear of persons wearing masks because their identity and their facial expressions were not recognizable. The parents, physicians, and teachers surveyed also reported exclusion, stigmatization, and aggressive behavior towards children who cannot wear masks for medical or psychological reasons [97]. The results of the study clearly show that it is not necessarily the protection measures themselves that have significantly impaired the psychological well-being of many children but rather the frequent pressure of conflicting expectations in the social environment.

According to [72], the everyday life of people in Germany in 2020 was dominated by two concerns: worry over financial troubles and fear of getting infected with COVID-19. Previous studies had found that parents‘ fears affect their parenting and ultimately the anxiety behavior of their children [98,99]. Clearly apparent here is the significance of the WHO’s One Health concept (described above) and its applicability to the dependence on the environment of children’s health and developmental opportunities. Regarding economic and social tensions, it will be particularly important in the future to extend the One Health concept to a ‘One Mental Health’ concept for psychological health and child protection. The call for zero violence against children is stated also in connection with the COVID-19 pandemic in the United Nations’ Sustainable Development Goals Report 2020 [100]. Here, it is important to pay special attention to children who were exposed to risk factors already prior to the pandemic. A survey in the last quarter of 2021 found a higher risk perception in the population regarding the spread of the virus and a concurrent increase in voluntary practicing of protective behaviors [101]. However, a study in 2020 in this context found that practices of protective behavior during the pandemic are dependent on educational level and that there is an association between a lower level of education and a lower likelihood of protective behaviors such as hand washing and reducing social contacts [102]. This, too, illustrates a health risk for children that is dependent on the environment.

A look at research studies on economic crises in the past makes it clear that economic stressors have a consistently negative impact on the physical and mental health of children and adolescents. Here again, the impact is greater on children with higher health and social risk factors than on children from supposedly more secure circumstances. Ajduković et al. [103] found that social support from family and friends in difficult economic times is a protective factor regarding child maltreatment for mothers with low socioeconomic status. However, since the start of the pandemic, precisely this social support has been only very limited or not possible at all. Moreover, a recent study in Australia demonstrated that in the course of the pandemic, covering the cost of basic needs, such as food and rent, became such an overriding everyday concern that in a survey of families with children rated at risk of maltreatment before the pandemic “the impact of the pandemic on the well-being of children was not a prominent theme for parents” [104] (p. 5).

It will be essential in the future to conduct studies on the long-term economic and social consequences of the COVID-19 pandemic for children and adolescents in order to identify influencing factors and to address needs that have changed in the areas of children’s health and support from child protective services. The review of the literature presented above does not claim to provide a conclusive description of economic and social policy factors. Moreover, the previous study findings have only limited applicability today, as there were no pandemics in recent decades with such comprehensive effects in a comparable global infrastructure. Additionally, because the pandemic is now ongoing, regarding the resulting social consequences only case examples of conflict areas can be described and analogous conclusions derived.

The Competence Area Mental Health Prevention in the Competence Network Preventive Medicine Baden-Württemberg in cooperation with the Competence Center Child Abuse and Neglect Baden Württemberg (both funded by the Ministry of Science, Research and Arts Baden-Württemberg), will determine and analyze economic and social policy effects of the pandemic, with the aim of mental health prevention. Incorporating the WHO’s One Health approach will play an important role as previous studies have shown that the progressing climate change will have an impact on the mental health of children and adolescents not only due to the increased risk of the spread of infectious diseases but also due to change of the social, ecological, economic, and cultural environment [26]. In this context, in addition to the necessary anchoring of children’s rights in the constitution, measures must also be taken against the societal endangerment of child welfare, such as by the effects of impending climate disasters [105]. It is essential to consider children and adolescents in policy decisions pertaining to the current COVID-19 pandemic and to include the consequences of climate change for the coming generations in climate policy [106].

## Data Availability

Not applicable.
